# Glucagon-stimulated copeptin response in children: a proof-of-concept study

**DOI:** 10.3389/fendo.2026.1833629

**Published:** 2026-06-12

**Authors:** Rita Indirli, Federico Giacchetti, Eriselda Profka, Giulia Rodari, Iulia Petria, Maura Arosio, Claudia Giavoli, Giovanna Mantovani, Emanuele Ferrante

**Affiliations:** 1 Endocrinology Unit, Fondazione IRCCS Ca’ Granda Ospedale Maggiore Policlinico di Milano, Milan, Italy; 2 Department of Clinical Sciences and Community Health, Dipartimento di Eccellenza 2023-2027, University of Milan, Milan, Italy

**Keywords:** arginine-vasopressin deficiency (AVP-D), copeptin, glucagon stimulation test, growth hormone (GH) deficiency, polyuria-polydipsia syndrome

## Abstract

**Purpose:**

The diagnostic workup of polyuria-polydipsia syndrome (PPS) is not well-established in children and adolescents, and several non-osmotic copeptin-stimulating agents have been investigated. Glucagon stimulation test (GST) induced a robust copeptin response in adults, but data in children are lacking. This study aimed to investigate copeptin response to GST in children tested for suspected growth hormone (GH) deficiency.

**Methods:**

Twenty children (9 girls, age 10.3 ± 2.9 years) with no PPS underwent GST (30 µg/kg of glucagon i.m.). Plasma glucose, GH, cortisol, and copeptin were measured before and 60, 90, 120, 150, and 180 min after glucagon injection.

**Results:**

Median copeptin concentration was 4.1 pmol/L (IQR 3.3–6.7) at baseline and 10.6 pmol/L (5.4-17.9) at peak concentration (+87% at 150 min). No adverse events were recorded.

**Conclusion:**

GST is safe and effective at enhancing copeptin secretion in children with intact neurohypophysial function, and further studies are warranted to assess its accuracy in the differential diagnosis of PPS.

## Introduction

Polyuria-polydipsia syndrome (PPS) can be caused by primary polydipsia (PP), arginine-vasopressin deficiency (AVP-D), or arginine-vasopressin resistance (AVP-R). Establishing the correct diagnosis is challenging due to overlapping presentation. Baseline copeptin, if ≥21.4 pmol/L, can identify AVP-R with high accuracy. Conversely, it cannot differentiate AVP-D from PP. For this purpose, osmotic or non-osmotic provocative tests are required (1).

Osmotic tests include the water deprivation test (WDT) and the hypertonic saline test (HST). HST is the gold standard in adults (1) but is rarely used in children. Recently, Ciortea et al. reported that HST-stimulated copeptin <6.5 pmol/L had 100% accuracy in diagnosing AVP-D in children (2). Nevertheless, safety concerns remain, and recent reviews have stated that HST does not represent a viable option because of its unfavorable safety profile; consequently, it should be reserved for selected cases in highly specialized centers only (3, 4).

WDT is the gold standard in pediatric PPS, despite several drawbacks, too. First, it has never been validated in children, and its accuracy is limited due to overlapping laboratory parameters between (partial) AVP-D and PP. Additionally, it requires hospitalization and close supervision and may lead to dehydration and hypoglycemia (3, 5).

The observation that other stimuli can enhance AVP/copeptin release has fostered the development of non-osmotic tests. Insulin (6, 7), arginine (8, 9), and L-DOPA (10), alone or in combination (11, 12), have been tested in children with variable, non-definitive results. Glucagon, instead, has been tested in adults only, where it was reported to significantly increase copeptin in healthy volunteers and PP but not in AVP-D (13). Glucagon-stimulated copeptin had 95% accuracy in differentiating these two conditions.

The aim of this study was to assess copeptin response to the glucagon stimulation test (GST) in a cohort of children evaluated for short stature.

## Materials and methods

### Patient selection and procedures

This observational, cross-sectional, proof-of-concept study was conducted at the University Hospital “Fondazione IRCCS Ca’ Granda Ospedale Maggiore Policlinico” in Milan, Italy.

Participants were consecutively recruited among pediatric patients (age <18 years) evaluated for short stature and/or reduced growth velocity (14). After exclusion of other causes of growth impairment, patients with an arginine test suggestive of GH deficiency (GHD) underwent GST for diagnostic confirmation. Participants had no history of PPS or acute illness at the moment of evaluation.

All participants underwent GST (30 μg/kg of glucagon, maximum 1 mg i.m.) (15–17) between 8 and 9 a.m. after an overnight fast. No fluid restriction was required. Blood samples for measurement of glucose, GH, cortisol, and copeptin were drawn before and 60, 90, 120, 150, and 180 min after glucagon injection. Other routinary laboratory and clinical data were retrieved from medical records.

Sex-steroid priming was performed in prepubertal (Tanner stage I) boys aged ≥11 years and girls aged ≥10. Boys received i.m. testosterone enanthate 50 mg 1 week prior, and girls received oral estradiol valerate 2 mg once daily for 3 days prior to GH stimulation testing.

Study procedures were in accordance with the principles of the Declaration of Helsinki. The study was approved by the Milan Area 2 Ethics Committee (ID 1267). Written informed consent was obtained from parents or legal guardians for all participants.

### Hormonal assays

Serum copeptin was assessed using a commercially available automated sandwich immunoassay (B.R.A.H.M.S. Copeptin proAVP KRYPTOR, Thermo Fisher Scientific, Waltham, Massachusetts, USA). The immunoassay has a limit of detection of 0.69 pmol/L, a functional sensitivity of 1.08 pmol/L, and an interassay coefficient of variation <18%.

Serum GH was assayed using a chemiluminescence method (Immulite 2000, Siemens Medical Solutions Diagnostics, Los Angeles, CA) with a detection limit of 0.01 μg/L. The standard was calibrated to the WHO International Standard IS98/574. GH peak concentrations ≥8 µg/L were considered normal (18–20).

Serum cortisol was measured using the second-generation monoclonal immunoassay Elecsys Cortisol II (Roche Diagnostics, Mannheim, Germany, on CobaS E 602) with a limit of detection 1.5 nmol/L, a limit of quantitation of 2.0 nmol/L, an interassay coefficient of variation ranging from 1.9% to 10.1%, and an intra-assay coefficient of variation ranging from 1.5% to 5.4%. A cortisol peak concentration ≥136 µg/L was considered normal (21).

### Endpoints

The primary endpoint was the variation in copeptin concentrations from baseline following glucagon administration. The secondary endpoint was the association of copeptin with blood glucose, GH, and cortisol concentrations at different time points during GST.

### Sample size

According to previously published data (22), baseline copeptin levels in healthy adults were normally distributed with a mean value of 7.9 (SD 8.7) pmol/L and reached a mean peak concentration of 15.9 (SD 16) pmol/L 180 min after glucagon administration.

Accordingly, for the primary endpoint, 16 patients have to be included to reject the null hypothesis with a power of 0.9 and a probability of alpha error of 0.05.

### Statistical analysis

Distribution of quantitative variables was assessed by the Shapiro–Wilk test. Normally distributed variables were expressed as mean (SD), whereas variables with a skewed distribution were reported as median (IQR); qualitative variables were presented as absolute and percent frequencies.

Paired or unpaired *t*-test was performed to compare the means of normally distributed variables. Alternatively, the Mann–Whitney and Wilcoxon tests were used. Frequencies of qualitative variables were compared by Fisher’s exact test.

Repeated measures correlation analysis was conducted to quantify the association between paired variables (copeptin with each one of: glucose, cortisol, GH) recorded multiple times (6 time points) per study subject. A multilevel mixed regression model was conducted as a multiple regression test involving repeated measures (i.e., copeptin: dependent variable; glucose, GH, cortisol, and time: independent fixed effects; patient ID: random effect).

A two-sided *p*-value was considered statistically significant when ≤0.05. Analysis was performed using GraphPad Prism (version 10) and R software (version 4.1.0).

## Results

Twenty children were included (9 girls, age 10.3 ± 2.9 years; BMI 15.6 ± 2.3, BMI SDS −0.7 ± 1.2). Four had isolated idiopathic GHD confirmed by negative magnetic resonance imaging and normal functioning of other hypothalamus–pituitary axes. Among the 16 patients in whom GHD was excluded, the causes of short stature were as follows: constitutional delay of growth and puberty (*n* = 1), born small for gestational age (*n* = 1), idiopathic short stature (*n* = 13), and pubertal delay in Klinefelter syndrome (*n* = 1). Three patients were treated with cholecalciferol at the time of the test. No other concomitant therapy was recorded.

Cortisol responses to GST were normal in all participants. In three patients, cortisol concentrations were higher at baseline than at any other time points; in three other patients, the highest GH (but not cortisol) concentrations were observed at baseline as well, and in one more patient, both hormones showed utmost concentrations preceding glucagon injection. This was regarded as a stress response to venipuncture. However, among these patients, only two exhibited copeptin levels that were higher at baseline than in other samplings. GST was well-tolerated. Neither nausea and vomiting nor serious adverse events were reported.

### Copeptin response to GST

**Figure 1:**
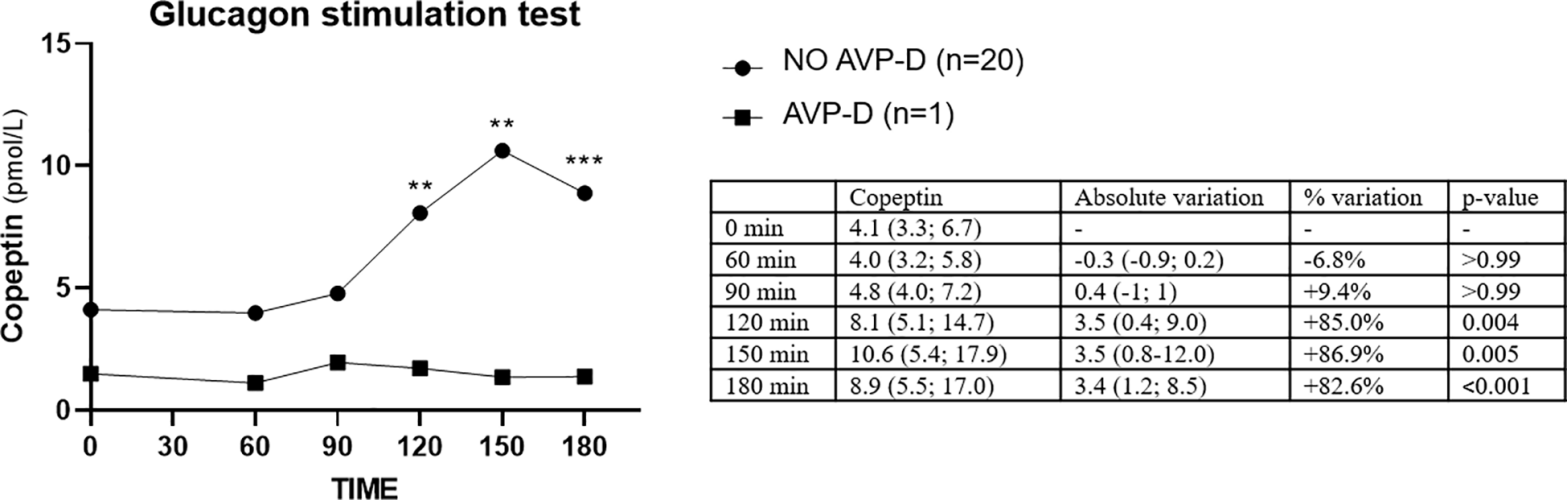
Median copeptin concentrations at different time points during the glucagon stimulation test in 20 short children with no AVP deficiency (no AVP-D, line and circles). The table on the right side reports median (IQR) copeptin concentrations (pmol/L), absolute (pmol/L) and percent variation and the *p*-value compared to baseline copeptin, in these subjects. The line with squares represents the only patient included with established AVP-D. **p < 0.01 vs. baseline; ***p < 0.001 vs. baseline.

Median baseline copeptin was 4.1 pmol/L (IQR 3.3; 6.7). During GST, a significant copeptin increase was recorded at 120, 150, and 180 min (*p* < 0.01 vs. baseline, **Figure 1**), with peak concentrations reached at 150 min (median 10.6, IQR 5.4; 17.9 pmol/L).

With the limitation of small sample size, no significant difference was observed in copeptin concentrations in patients with and without GHD (**Table 1**).

**Table 1:** Characteristics and copeptin response in patients with and without GH deficiency (GHD).

Variable	GHD *N* = 4	No GHD *N* = 16	*p*
Age (years)	9.6 (1.9)	10.4 (3.2)	0.62
Female *n* (%)	3 (75%)	6 (37%)	0.28
IGF-1 (µg/L)	132 (95.2; 177.8)	133.5 (80.5; 186.0)	0.95
IGF-1 SDS	−1.1 (−1.3; −0.4)	−1.1 (−1.5; −0.5)	0.81
Copeptin, baseline (pmol/L)	2.9 (2.3; 5.9)	4.2 (3.6; 7.6)	0.12
Copeptin, 60 min (pmol/L)	3.0 (2.4; 4.2)	4.3 (3.8; 6.0)	0.05
Copeptin, 90 min (pmol/L)	4.3 (2.1; 29.2)	4.8 (4.2; 7.2)	0.68
Copeptin, 120 min (pmol/L)	4.8 (2.6; 15.7)	9.6 (5.7; 14.7)	0.18
Copeptin, 150 min (pmol/L)	5.3 (3.5; 11.9)	15.1 (6.6; 19.8)	0.08
Copeptin, 180 min (pmol/L)	6.3 (4.1; 9.3)	10.5 (6.4; 26.9)	0.15

Quantitative variables are expressed as mean (SD) or median (Q1; Q3) as appropriate.

IGF-1, insulin-like growth factor 1; IGF-1 SDS, IGF-1 expressed as standard deviations from age-specific mean values.

### Correlation study

In **Table 2**, changes in copeptin, cortisol, GH, and glucose concentrations along the GST are reported. Copeptin was directly associated with cortisol (*r* = 0.39, *p* < 0.001) and GH (*r* = 0.42, *p* < 0.001) and inversely associated with glucose (*r* = −0.36, *p* < 0.001) in repeated-measures correlation analysis. However, in a multilevel mixed-effects regression model, copeptin was significantly associated with cortisol only (*β* = 0.375, *p* = 0.01), but not with GH and glucose.

**Table 2:** Copeptin, cortisol, growth hormone (GH), and glucose plasma concentrations recorded during the glucagon stimulation test.

Time-point	Copeptin (pmol/L)	Cortisol (µg/L)	GH (µg/L)	Glucose (mg/dL)
Baseline	4.1 (3.3; 6.7)	101 (89; 136)	2.1 (0.5; 5.0)	82 (76; 88)
60 min	4.0 (3.2; 5.8)	70 (61; 88)	0.4 (0.2; 0.8)	116 (87; 126)
90 min	4.8 (4.0; 7.2)	84 (55; 123)	1.0 (0.2; 3.4)	76 (68; 80)
120 min	8.1 (5.1; 14.7)	105 (66; 130)	4.8 (2.2; 12.7)	65 (58; 72)
150 min	10.6 (5.4; 17.9)	96 (92; 173)	6.0 (2.8; 7.7)	73 (66; 55)
180 min	8.9 (5.5; 17.0)	131 (93; 181)	2.9 (1.6; 5.3)	75 (70; 80)

All variables are presented as median (Q1; Q3).

### GST in AVP-D

GST was performed also in an 11.5-year-old female patient with well-established AVP-D. Copeptin remained low from baseline (1.5 pmol/L) throughout GST (peak 2.0 pmol/L, **Figure 1**).

## Discussion

In this study, glucagon administration significantly increased copeptin serum concentrations in children without PPS. Thus far, similar data have been available in adult cohorts only. Atila et al. reported that copeptin increased by 172% in 22 healthy adults and by 364% in 10 patients with PP after glucagon administration (13). Consistently, Lewandowski et al. documented a robust increase in copeptin concentrations 150 and 180 min in healthy subjects (22). The response obtained with glucagon was more pronounced than previously reported with other non-osmotic agents (3), so GST appears to be a promising test. Actually, the median copeptin increase observed in children was not as marked (+87% at peak). However, our sample is heterogeneous and not fully representative of a healthy pediatric population, thus limiting generalizability to children with polyuria-polydipsia. By revising previous studies in pediatric cohorts, glucagon appears to be a more robust stimulant than arginine (8, 9), similar to insulin (6, 7), and weaker than L-DOPA (10), combined arginine–levodopa/carbidopa (12) or arginine–insulin (11) stimulation tests.

Outside the study protocol, we also reported a blunted response of copeptin to GST in a single patient with AVP-D. Although no conclusions can be drawn, this observation paves the way for further studies to assess the performance of GST in differentiating AVP-D from PP.

GST is widely available and well-tolerated. Consistently, we reported no adverse event. Of note, although nausea/vomiting is reported with glucagon (13), it did not occur in our cohort. Nausea/vomiting is a confounding factor in copeptin stimulation tests, since it can falsely increase AVP/copeptin release especially in partial AVP-D (23).

The precise mechanism by which glucagon stimulates AVP/copeptin, as well as GH and cortisol, remains largely unexplained. The drop in blood glucose concentrations is crucial to GH—but not cortisol—response (24) and may be linked to copeptin release as well, either as a direct effect or through reduction of the hypothalamic somatostatinergic tone (25). A correlation between glucose drop and copeptin increase upon glucagon stimulation was previously reported (26). Actually, we failed to confirm the significant association between glucose and copeptin concentrations, although this type of analysis is not sufficiently accurate to draw mechanistic conclusions. Secondly, glucagon-related hyperglycemia may act through plasma osmolality increase. Lastly, it may be a direct effect exerted through glucagon receptors in the hypothalamus/neurohypophysis or represent a stress response. Interestingly, in our study, copeptin was independently associated with cortisol during GST, which is consistent with the correlation of peak copeptin with ACTH and cortisol initially reported by Lewandowski et al. (22). This observation could reflect the well-known stimulation of corticotrophs from AVP through V1b receptors and, eventually, the release of cortisol from the adrenal cortex. However, other studies failed to confirm this association (27), which could just reflect covariation in response to the same stimulus, rather than causality.

In conclusion, we demonstrated that glucagon can act as an effective, non-osmotic, copeptin stimulant in children with intact posterior pituitary function. Further studies in larger and more balanced cohorts of children are warranted to test its performance in the differential diagnosis of PPS.

## Data Availability

The raw data supporting the conclusions of this article will be made available by the authors, without undue reservation.
